# Deriving Early Citrus Fruit Yield Estimation by Combining Multiple Growing Period Data and Improved YOLOv8 Modeling

**DOI:** 10.3390/s25154718

**Published:** 2025-07-31

**Authors:** Menglin Zhai, Juanli Jing, Shiqing Dou, Jiancheng Du, Rongbin Wang, Jichi Yan, Yaqin Song, Zhengmin Mei

**Affiliations:** 1 College of Geomatics and Geoinformation, Guilin University of Technology, Guilin 541006, China; 2120222042@glut.edu.cn (M.Z.); 2003080@glut.edu.cn (J.J.); 1020242101@glut.edu.cn (J.D.); 2 Shandong Mingjia Survey and Surveying Co., Ltd., Zibo 255086, China; 17852318111@163.com; 3 College of Mechanical and Control Engineering, Guilin University of Technology, Guilin 541006, China; 2019178@glut.edu.cn; 4 Guangxi Academy of Specialty Crops, Guilin 541004, China; wrongpiano@163.com (Y.S.); mzm077@126.com (Z.M.)

**Keywords:** crop load, citrus identification, multigrowth period prediction, lightweight networks, yield estimation models

## Abstract

Early crop yield prediction is a major challenge in precision agriculture, and efficient and rapid yield prediction is highly important for sustainable fruit production. The accurate detection of major fruit characteristics, including flowering, green fruiting, and ripening stages, is crucial for early yield estimation. Currently, most crop yield estimation studies based on the YOLO model are only conducted during a single stage of maturity. Combining multi-growth period data for crop analysis is of great significance for crop growth detection and early yield estimation. In this study, a new network model, YOLOv8-RL, was proposed using citrus multigrowth period characteristics as a data source. A citrus yield estimation model was constructed and validated by combining network identification counts with manual field counts. Compared with YOLOv8, the number of parameters of the improved network is reduced by 50.7%, the number of floating-point operations is decreased by 49.4%, and the size of the model is only 3.2 MB. In the test set, the average recognition rate of citrus flowers, green fruits, and orange fruits was 95.6%, the mAP@.5 was 94.6%, the FPS value was 123.1, and the inference time was only 2.3 milliseconds. This provides a reference for the design of lightweight networks and offers the possibility of deployment on embedded devices with limited computational resources. The two estimation models constructed on the basis of the new network had coefficients of determination R^2^ values of 0.91992 and 0.95639, respectively, with a prediction error rate of 6.96% for citrus green fruits and an average error rate of 3.71% for orange fruits. Compared with network counting, the yield estimation model had a low error rate and high accuracy, which provided a theoretical basis and technical support for the early prediction of fruit yield in complex environments.

## 1. Introduction

Citrus (family Rutaceae, genus Citrus) is the world’s number one fruit crop. China has the world’s largest area of citrus cultivation and the highest output; the citrus industry has become a pillar of southern China and an important factor in economic growth [1,2,3]. In citrus production, early yield estimation refers to the technique of predicting the final crop yield by analyzing relevant phenotypic characteristics such as the developmental status of flowers and the process of fruit formation, combining the correlation between these characteristics and yield, and applying quantitative models or data analysis techniques to predict the final yield of the crop. Rapid and accurate early yield prediction enables producers to understand in advance the relationships among flowering intensity, fruit loading, and fruit quality; this can be used to guide field management and is an important basis for farmers and producers to plan and allocate human and economic resources and to formulate marketing strategies [4,5,6].

In recent years, deep learning techniques have shown excellent advantages in image detection, and the fast and accurate ability of target detection networks has demonstrated good performance in crop estimation [7]. Currently, fruit yield estimation research using deep learning techniques is mainly based on image features of a single growth period, such as the flowering stage [8,9], green fruit stage [10,11], or ripening stage [12,13]. Predictions based on fruit blossoming determine the potential yield by analyzing the number, morphology, and distribution of flowers for pollination and early yield prediction. Although effective yield estimation can be achieved, this period is susceptible to climate changes, pests, and diseases, and the timeliness and accuracy of data collection is also an important factor; failing to obtain the data at the optimal time may lead to bias when predicting the overall yield. Predictions based on the characteristics of a single period of fruit greening or ripening are strongly affected by environmental conditions. Fruit in the greening period may ripen early or late due to changes in natural conditions or management measures, and insufficient soil moisture and nutrient supply may also lead to slow fruit growth, thus affecting overall yield prediction. Crop growth is a continuous time accumulation process, and only by combining data from multiple growing periods can the key drivers of growth processes and complexity be revealed. Therefore, combining multiple growth period data for early crop yield estimation can not only significantly improve the timeliness and accuracy of the prediction but also provide timely feedback of the estimation results for field management, thus realizing precise control of flowering intensity, fruit distribution density, and fruit quality, which is highly practical for the development of precision agriculture.

To construct a more accurate model for early fruit yield estimation to overcome the limitations caused by single growth period data prediction, Li et al. [14] utilized the YOLOv5 network to monitor the climatic period of oil tea fruits (buds, flowers, and fruits), which provided a theoretical reference for further research on the changes in oil tea fruit characteristics and accurate yield estimation and monitoring. Zhang et al. [15] established a multiobjective detection method based on the YOLOX model for monitoring the growth of tomatoes during the whole growth period in a real greenhouse environment, which provides a solution for the efficient monitoring of tomato flowers and fruits in complex scenarios. However, the complexity of multigrowth period data places greater requirements on the target detection network, and the large differences in multigrowth period data in complex scenes significantly increase the computational burden when coping with high-resolution images or multitarget scenarios, leading to difficulties in real-time detection. The existing detection network model used in the abovementioned joint multigrowth period data for crop yield estimation and monitoring is large, currently remains only in the laboratory stage, and cannot yet be deployed on portable devices with limited computational resources. Therefore, when dealing with different kinds of complex images, a lightweight model with detection accuracy becomes the key to improving detection performance. Traditional convolutional neural networks use convolutional kernels to generate feature maps at different scales, which leads to an increase in the computational cost of the model; how to reduce the computational loss when extracting features and minimize the loss of model performance has yet to be studied in depth. Factors such as light changes and branch and leaf occlusion in modern orchards challenge the network in detecting fruit crops, and the possibility of a lightweight detection head designed on the basis of the group normalization idea, which can reduce the problem of gradient disappearance in the training process and at the same time improve target localization and accuracy, remains to be further verified.

At present, relying solely on the number of fruits recognized by the deep learning network directly results in large yield estimation errors [16,17,18,19]; to reduce the yield estimation error, many scholars have used the network to recognize the counts of the joint regression method to carry out research. Mirbod et al. [20] used the Fast-RCNN network to recognize the manual counts of the linear fitting for apple production. Li et al. [21] fitted the YOLOv7 network counts with manual counts to establish a longan yield estimation model, and their results showed that the model prediction result after linear fitting was R^2^ = 0.995 with an average error of only 2.99%. Gao et al. [22] utilized the YOLOv4-tiny network to estimate longan yields via a linear regression model with manual counts and a linear regression model. The authors constructed a linear regression model to estimate apple yield, with an R^2^ = 0.9875 and an average error rate of 5.24%. The joint network identification and regression method can effectively reduce the counting error and improve the accuracy of fruit yield estimation; however, the current study is limited to the single growth period of the fruit counting joint regression method for statistics, and the number of detections in each growth period of the fruit as well as the correlation degree with the final yield still need further research and analysis to further improve the accuracy of the early yield estimation model.

In summary, this work takes the Pengyu Brothers Citrus Demonstration Base in Guilin city, Guangxi Province, and the Citrus Plantation Practice Base in Lingchuan County as the study areas, and collects close-up images of citrus in different growing periods (March, October, and December 2023) as the experimental samples for experimentation. The main research content of this paper is as follows:

(1) The image data of the citrus blossoming period, green fruit period, and fruiting period in multiple growth periods are collected, a data enhancement strategy is used to increase the number of images, and a feature dataset of citrus fruits in multiple growth periods is constructed.
(2) A new YOLOv8-RL network is constructed by combining the multigrowth period data with multiple strategies. Set up comparative experiments of different categories to verify the detection performance of the new network.
(3) Research and regression analysis are performed on the number of detections and the degree of correlation between each growth period of the fruit and the artificial yield to improve the accuracy of the citrus early yield estimation model and to verify the accuracy and applicability of the model.

## 2. Materials and Methods

### 2.1. Image Acquisition

The close-up image collection site for multiple citrus growing periods is located in the Pengyu Brothers Citrus Demonstration Base in Gongcheng County, Guilin city, Guangxi Zhuang Autonomous Region (24°51′42.07″ N, 110°49′7.06″ E), and Changling Shang Citrus Plantation Practice Base in Tanxia town, Lingchuan County (23°56′41.26″ N, 115°32′26.92″ E). The sites are suitable for the growth of fruit trees such as Merkert, Citrus sinensis, and sugar orange. After the tree species, tree age, slope, and other factors were considered, citrus fruit trees were randomly selected from the two study areas for image acquisition. The collection species is Merkert, the tree is about 7 years old, the image acquisition time for 2023 included the citrus flowering collection time from March 15 to 17, the green fruit period from October 10 to 13, and the ripening period from December 20 to 24. To improve the model’s resilience and versatility, this research utilized Huawei Mate40 Pro and Nova mobile devices to capture images simultaneously. The images were taken from different viewpoints (e.g., upward and downward angles) at two different research sites. Real-world images were obtained under a range of growth conditions, encompassing sparse, dense, occluded, and overlapping scenarios. A total of 760 images of citrus fruits were collected for the whole period, including 234 images at the flowering stage, 289 images at the green fruit stage, and 237 images at the ripening stage, in JPG format, with a resolution of 4096 × 3072 pixels per image.

### 2.2. Image Enhancement and Dataset Segmentation

**Figure 1:**
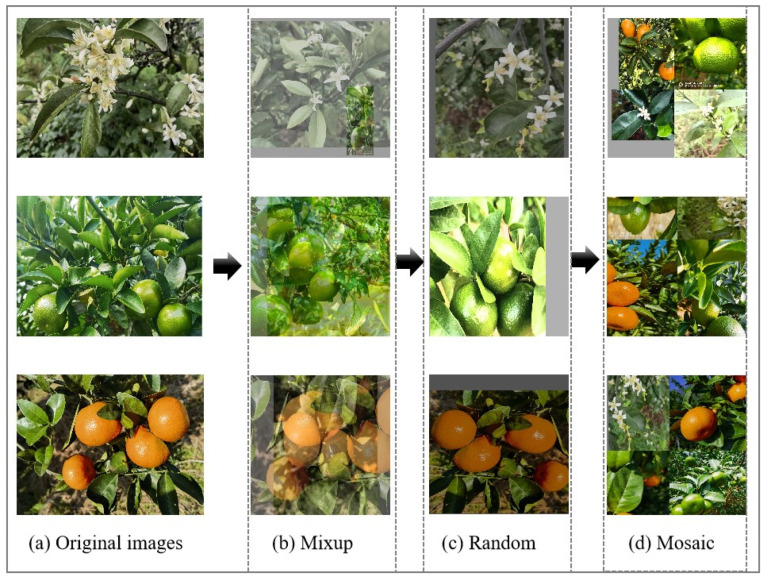
Citrus multigrowth period image data: (**a**) original citrus images at the flowering, green fruit, and ripening stages; (**b**) mixup enhancement method; (**c**) random enhancement method; (**d**) mosaic enhancement method.

In the natural environment, disturbance factors such as citrus fruit tree stem and leaf shading, weather, and light directly affect the detection rate of the network. To improve the robustness of the model, the model’s ability to learn the images of citrus fruits during the whole growth period was enhanced, and the overfitting phenomenon caused by too small a dataset was prevented [23]. In this study, from 760 images, 250 clear and representative images were selected for data augmentation, of which 50 images were in the flowering stage, 100 images were in the green fruit stage, and 100 images were in the ripening stage. The data preprocessing process was as follows: first, the original images were manually labeled with the image annotation software LabelImg (v1.5.1), and then mixed data enhancement of the images was performed via gamma change, image equalization, the mixup method, and the mosaic method; the effect of the images after data enhancement is shown in Figure 1. After data enhancement, 3250 images were finally obtained as the total dataset for this study.

**Figure 2:**
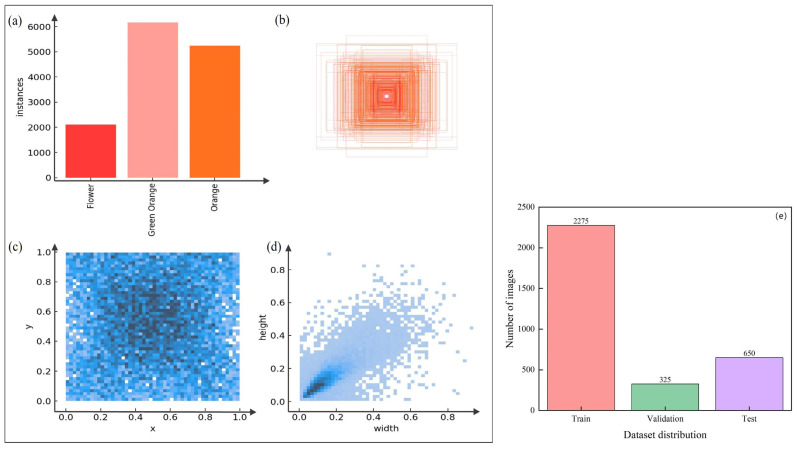
Distribution of citrus multigrowth period data: (**a**) number of samples of different categories of citrus, (**b**) distribution of target frame scales, (**c**) distribution of target frame centroids, (**d**) target frame aspect ratios, and (**e**) number of samples in the dataset.

In this study, the data were stored according to the Microsoft public dataset MS COCO format, and 3250 data-enhanced images were randomly divided into training, validation, and test sets at a ratio of 7:1:2. A total of 18,905 samplelabels were captured in the dataset, as seen from the analysis in Figure 2, in which the training set consisted of 2275 images, of which 13,186 labels were captured, with a comparable number of green citrus and ripe citrus and a smaller percentage of labels in the blooming stage, which was comparable to the proportional number during data screening. After data enhancement, the centroids of the detection frames of all the detected objects were more in the middle part of the images, centered, and the width and height of the detection frames were close to 1, indicating that the detection frames were close to square. Table 1 shows the specific division of the dataset.

## 3. Research Methodology

In this paper, a more accurate model for early citrus fruit yield estimation is proposed by combining multigrowth period data with the improved YOLOv8. First, we collect close-up images of citrus fruits during the flowering, green fruiting, and ripening periods and use data enhancement strategies to expand the number of images to construct a citrus multigrowth period dataset. Second, we improve the YOLOv8 detection network to propose a new model that is lighter and more accurate, validate the comparative analysis of the actual detection performance of the new network, and set up ablation experiments to validate the effectiveness of the improvement strategies. Third, we establish a regression model based on the number of different growth period counts identified by the network and the manual counts. Finally, regression models are established on the basis of the counts of different growth periods identified via the network and manual counts to analyze the differences in the number of identified networks, the real number of fruits, and the number of fruits predicted by the regression model and to reverify the accuracy and applicability of the predictions of the constructed regression model.

### 3.1. YOLOv8 Modeling

**Figure 3:**
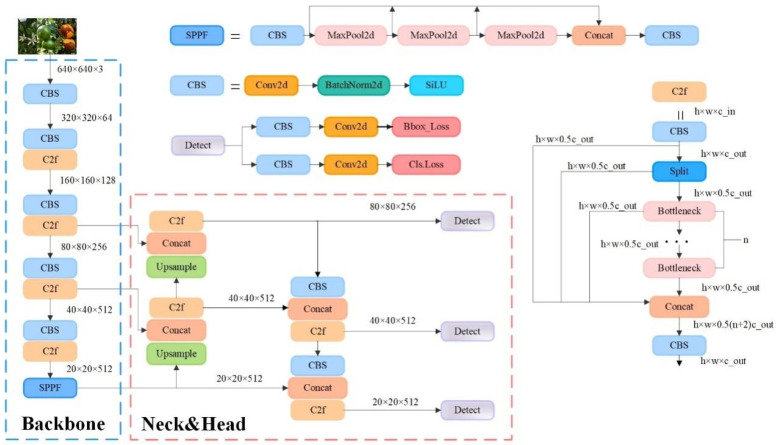
YOLOv8 network structure diagram.

YOLOv8 (you only look once version 8) is the latest version of the YOLO series proposed by Ultralytics on 10 January 2023; it inherits the high efficiency and real-time performance of YOLO while maintaining the advantages of the engineering simplicity and ease of use of YOLOv5 [24]. YOLOv8n/s/m/l/x versions are provided according to different hardware resources and application requirements. YOLOv8n is the version with the smallest model and the fastest speed, and its network structure is shown in Figure 3. The backbone mainly consists of three parts, namely, CBS (Conv2d, BatchNorm2d, SiLU), C2f, and SPPF, where the C2f module improves the feature expression and detection performance via cross-layer fusion, making it possible to improve the feature expression and detection performance so that the network captures multiscale information and details; the SPPF module divides the feature map into multiple regions for the pooling operation to improve the robustness of the network. The neck part replaces the C3 module in the predecessor version, YOLOv5, with the C2f module, which enables the network to have richer gradient streams through more residual connections. The head part is composed of the detect module, which can detect targets of different scales, and adopts the mainstream decoupled head structure, which separates the classification and detection heads, so that they can be carried out separately to enhance the effect.

### 3.2. Multistrategy Construction of a New YOLOv8-RL Network

**Figure 4:**
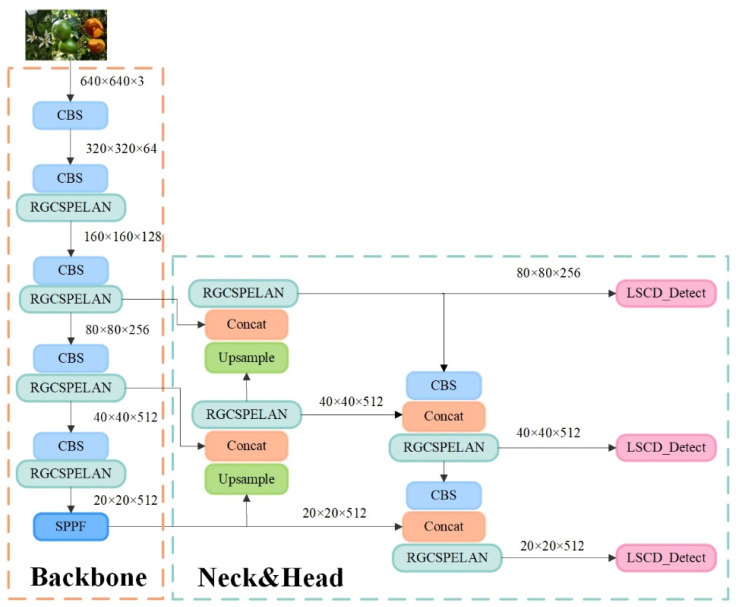
Improved YOLOv8-RL new network structure diagram.

To optimize the speed and model size, YOLOv8n reduces the model complexity, leading to poor performance when dealing with complex background or dense object scenes in citrus orchards and limitations when dealing with similar and overlapping targets; there is room for further optimization of the model size and the number of parameters, even though the model size is small. In summary, this study explores ways to improve the new network based on the YOLOv8n model for the accurate detection of full-growth-period images in complex scenes in citrus orchards and further shrinks the model while ensuring detection accuracy and prediction speed for deployment in resource-limited devices. The improved network model is shown in Figure 4. The new network uses the RGCSPELAN module to replace the C2f module, which is used to reduce model complexity and the number of parameters, and a lightweight detection head LSCD, is used to replace the detect module in the original model, which further reduces the number of parameters while guaranteeing detection head localization and classification performance.

#### 3.2.1. Enhanced Citrus Feature Extraction and Optimization Calculation Using the RGCSPELAN Module

In actual citrus orchards, complex natural environments, such as weather factors, branch and leaf shading, fruit backgrounds, and other redundant information, affect fruit detection accuracy. Citrus flowers are small and dense, with severe overlap and adhesion of flower clusters; fruits grow densely during the green fruit stage, with overlapping occlusion and presenting a green color similar to the color of leaves; and the surface of fruits tends to be smooth during the ripening stage, with overexposure of the image and severe overlapping of the fruits. Currently, traditional convolutional neural networks (CNNs) rely on many convolutional kernels to generate feature maps at different scales to capture feature information in complex images, which leads to high computational costs and many model parameters; however, many redundancies still exist in the actual feature maps, so the process of generating these feature maps may not require high computational overhead [25].

**Figure 5:**
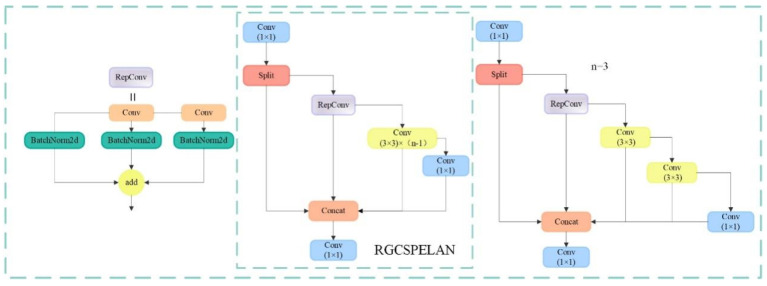
Structure of RGCSPELAN.

On this basis, this study uses a simple linear transformation, i.e., the standard convolutional block, to generate fewer basic feature maps to reduce the computational and parametric quantities when extracting features and discarding the commonly used BottleNeck module in the original model, YOLOv8. To compensate for the performance loss caused by discarding the residual block, RepConv is used on the gradient circulation branch to enhance the feature extraction and gradient circulation ability. RepConv uses a multiconvolutional kernel configuration in the training phase to enhance the feature extraction and gradient circulation capabilities of the network. With this design, gradient delivery and feature representation can be effectively improved. In addition, in the inference phase, the RepConv module is able to fuse multiple convolutional kernels into one equivalent convolutional kernel, which significantly reduces the computation and memory footprint [26]. The dual benefits of performance enhancement and computational optimization are realized, ensuring the efficiency and reliability of the network in practical applications. With the concept of the composite model scaling method and efficient layer aggregation network ELAN in the YOLOv7 [27] network, the module is redesigned, and the structure of the RGCSPELAN module is shown in Figure 5, where the scaling factor is adjusted by the split operation after standard convolution as a way of controlling the size of the module, which can take into account both the small model and the large model. A branch subsequently enters the RepConv module for feature extraction, and the size of n is adjusted to determine the number of 3 × 3 convolutions. Then, the standard convolution is performed again and ultimately spliced with another branch after the split operation to complete the fusion of feature maps to achieve better performance in the detection task.

#### 3.2.2. Lightweight Detection Head LSCD Design for Accurate Positioning of Citrus Targets

To further improve the model’s ability to detect citrus feature information at different scales and reduce the computational cost, in this study, the number of parameters in the head part of the detector module in the YOLOv8n network model accounts for approximately 30% of the total number of parameters, which significantly increases the computational overhead when performing multitask detection and multiscale prediction. On this basis, this study uses group normalization (GN), which combines the advantages of batch normalization (BN) and layer normalization (LN), to improve the computational efficiency of the network. GroupNrom improves the computational efficiency of the network in terms of localization performance by normalizing each feature group, which improves the training stability of the network and reduces the vanishing or exploding gradient problem that may occur during the training process. In addition, it reduces the internal covariate bias, which enables the network to better capture the location of the target features, thus improving the localization accuracy. In terms of classification performance, it can enhance the ability of the classification head to identify different classes of features and is insensitive to the batch size, and it can provide a more consistent normalization effect during the training process, which gives the model better generalizability under different training conditions. GroupNorm has been shown in FCOS (Fully Convolutional One-Stage Object Detection) to improve the performance of detection head localization and classification [28]. The group normalization formula is shown in (1):

(1) $$GroupNorm(x_{i})=\gamma \times \frac{x_{i}-\mu_{g}}{\sqrt{\sigma_{g}^{2}+\varepsilon }}+\beta$$

where *x_i_* is an element in the input feature map; *γ* is the scaling factor; *β* is the offset; $\mu_{g}$ and $\sigma_{g}^{2}$ are the within-group mean and variance, respectively; *ε* is a constant that prevents division by zero; and *g* denotes that the current normalization is performed on the g-th group.

**Figure 6:**
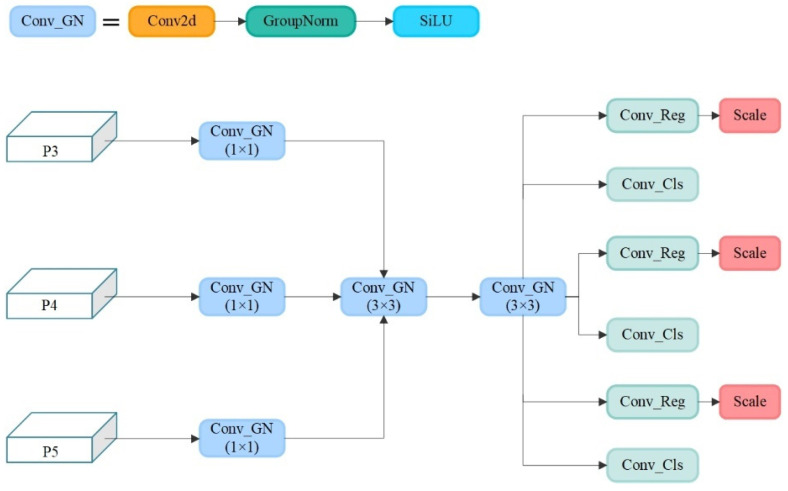
LSCD structure.

The structure of the lightweight detection head LSCD is shown in Figure 6. The Conv_GN module consists of standard convolution, group normalization, and the SiLU activation function. For the network after the P3, P4 and P5 layers, the obtained feature maps of different scales are first input into the Conv_GN, and then, using the idea of shared convolution, the shared Conv_GN (3 × 3) module is shared to efficiently extract the spatial features of the image; at the same time, the number of parameters is further reduced to reduce the memory occupation and computational burden. Moreover, while using shared convolution, to address the problem of the inconsistent scale of the targets detected by each detection head, the feature map is scaled via the scale layer to better match the actual scale of the targets. Combining the shared convolution and scale layers can make the network more flexible and robust, and can increase the accuracy of target localization and classification.

### 3.3. Experimental Platform and Parameterization

The experiment utilized the Windows 10 operating system on hardware comprising an i7-9800K@3.80 GHz CPU, 32 GB RAM, and an Nvidia GeForce RTX2060 graphics card. PyCharm (3.9) served as the compiler, Python version 3.10 was employed, and the PyTorch 2.2.1 development framework was utilized. For parallel computing, CUDA version 12.1 was applied, cuDNN version 11.8 for deep neural network GPU acceleration, and OpenCV version 4.4.5 for image processing. Model initialization parameters involved training from scratch without loading pre-training weights, with model training acceleration achieved by adjusting CPU thread numbers. Network hyperparameters included an input image size of 640 pixels × 640 pixels and a batch size of 16. Stochastic Gradient Descent (SGD) was used for model optimization, with an initial learning rate set at 0.001, a momentum factor of 0.937, and a weight decay factor of 0.0005. The model underwent 300 iterations, saving weights every 10 epochs, and ultimately selecting the best.pt weights for verification and testing.

The experiment assessed the model performance based on key indicators from the COCO dataset, such as accuracy (P), recall (R), average precision (mAP), F1 score (F1), model parameters, floating-point operations, model size, and frames per second (FPS) value. The determination coefficient R^2^ was employed as the evaluation metric for the production model.

The formulas of the above evaluation indices are shown in Equations (2)–(6):

(2) $$P=\frac{TP}{TP+FP}$$

(3) $$R=\frac{TP}{TP+FN}$$

(4) $$\mathrm{mAP}=\frac{\sum_{1}^{N}\int_{0}^{1}P(R)\mathrm{dR}}{N}$$

(5) $$F1=\frac{2\times P\times R}{P+R}$$

(6) $$R^{2}=1-\sum_{i=1}^{N}{\left(y_{i}-{\overset{`}{y}}_{i}\right)}^{2}{\left(\sum_{i=1}^{N}{\left(y_{i}-\overset{↼}{y}\right)}^{2}\right)}^{-1}$$

where TP denotes the positive sample predicted by the model, which refers to the number of correctly identified citrus full-growth stage labels (flowers, green fruits, and orange fruits); FP denotes the negative sample predicted by the model, which refers to the number of incorrectly identified citrus labels; FN denotes citrus labels that were not correctly detected; and N stands for the number of categories. $y_{i}$ represents the true value of the sample, ${\overset{`}{y}}_{i}$ is the precision of the predicted value of the sample, and $\overset{↼}{y}$ is the average of the true values.

## 4. Results and Discussion

### 4.1. Comparison of Different Network Models

To verify that the improved YOLOv8-RL new model is lightweight while still considering the accurate recognition ability of citrus full-growth period images, in this study, the current mainstream small model detection networks and the whole series of YOLOv8 networks as well as the current latest target detection networks are selected for comprehensive comparative analysis, including YOLOv5s, YOLOv7-tiny [27], YO LOv8n/s/m/l/x [24], YOLOv9t [29], and YOLOv10n [30]. All the network models are trained with the same dataset, no official default weights are loaded, and all the parameters are kept consistent. The weights with the highest accuracy were selected after the training was completed, and the comprehensive evaluation was performed on the same test set with a total of 650 citrus full-growth period images. In this work, FPS is calculated as follows: when BatchSize = 1, 1000 ms is divided by the sum of the image preprocessing time, inference time, and postprocessing time to obtain the final result. After complete testing, the performance metrics of the above target detection model are shown in Table 2 below, and all the metrics are the average accuracies of the three classifications of citrus (blossom, green, and orange fruit).

The analysis in Table 2 shows that YOLO5s still has good performance in long-term image detection in citrus. While it has high precision, the parameters, floating-point operation times, model size, and other indicators are still not inferior to those of the current mainstream detection network; however, owing to the long reasoning time and relatively low FPS value, it has lost the advantage of real-time detection. YOLOv7 tiny is a new detector proposed by the Alexey Bochkovskiy team in July 2022 [27]. The accuracy of the model in the test set is 97% mAP@.5. The index also has 96.6% performance. Although the F1 value reaches 0.95, the parameters, computational complexity, and model size are far lower than those of the optimal model. Second, as an improved version of the Yoov5 network, the YOLOv8 [24] series network not only maintains excellent performance but also enhances the model reasoning time and the efficiency of real-time detection. For the citrus dataset, the whole YOLOv8 series network has ideal performance. It can be seen from the data analysis in the table that with increasing depth and width of the network, the detection performance of the network is also gradually enhanced, but the consequent drawback is the high cost of computing resources. Taking YOLOv8n and YOLOv8x as examples, the detection accuracies for mAP@.5 and F1 values differ by 3.1%, 3.6% and 4%, respectively, but the parameter quantity, floating-point operation, and model size of the former are much smaller than those of the latter. Although YOLO8x has the highest detection performance, its high computational cost and large model proportion are not suitable for the current real-time detection requirements. When the parameters of the YOLOv8n model are moderate, the reasoning time and FPS value have better performance. YOLOv9t [29] and YOLOv10n [30] are the latest detection networks published at present. According to the data analysis in the table, although they have good performance in terms of the parameter quantity and model size in the citrus dataset test, each index has little advantage in citrus detection.

Finally, the best performing YOLOv8n is selected for a comprehensive comparison with the YOLOv8-RL proposed in this study, and the analysis shows that the performance of the improved network is close to that of the original network in terms of indicators such as P, mAP@.5, and the F1 value, which indicates that the improved network has no loss of detection accuracy. Second, the new network, YOLOv8-RL, has a decrease in the number of parameters by 50.7%, the number of floating-point operations is reduced by 49.4%, and the model size is only 3.2 MB, which is approximately one-half of the original model. In addition, the inference time of YOLOv8-RL is only 2.3 ms, and the FPS real-time detection frame rate is 123.1, which are both better than those of the original network. In summary, the experimental results show that the improved YOLOv8-RL network still maintains high detection performance after a large number of network parameters are reduced to lighten the network, achieves high detection accuracy with minimum model volume, low computational cost and high detection efficiency, and is more suitable for edge detection devices with scarce computational resources, which provides technical support for the application of the lightweight network to real-time citrus detection.

**Figure 7:**
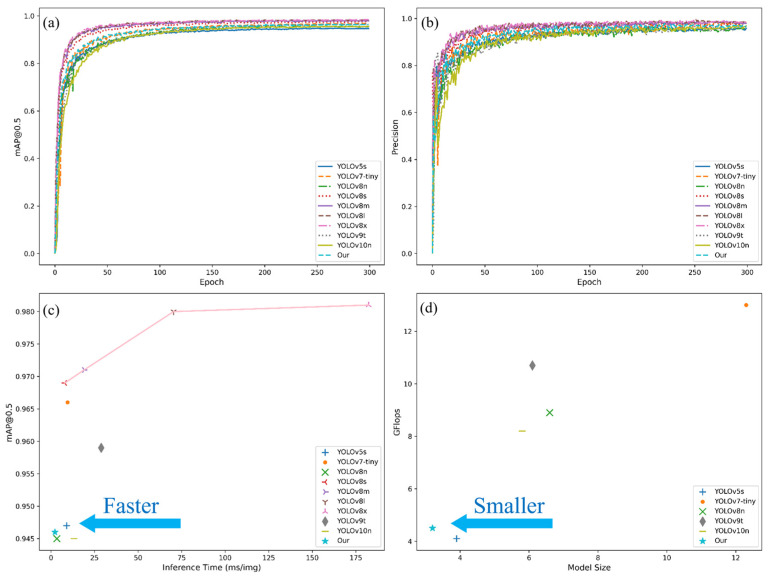
Network performance graph. (**a**) Trends in detection accuracy of different models during training. (**b**) Accuracy performance of different models during training. (**c**) Comparison of different models in terms of inference speed and detection accuracy. (**d**) Comparison of different models in terms of inference speed and detection accuracy.

To analyze the differences in network performance more intuitively, the most important mAP@.5 and accuracy P metrics are selected for visual analysis in this study, and all network models are shown in Figure 7a,b after 300 iterations. The analysis shows that all the involved detection networks start to converge gradually after 100 epochs and reach a smooth trend. The large model has excellent performance for citrus detection in complex environments, with the advantages of network width and depth, and has better performance with the same number of training times. YOLOv8n and the YOLOv8-RL lightweight model proposed in this study have similar detection performances. Figure 7c,d show the model inference time-mAP@.5 scatterplot and the model size floating-point operation count scatterplot, respectively. Figure 7c shows that YOLOv8-RL has the shortest inference time while considering the high detection performance. Figure 7d shows that the YOLOv8-RL network is not only the optimal model with a small model size but also has the lowest computational expenditure.

### 4.2. Comparative Analysis of Detection in Complex Scenarios over the Full Growth Period

Given the complexity of the orchard environment under natural conditions, the degree of light and darkness, the shading interference of fruit tree branches and leaves, and the prevalence of the overlapping fruit shading phenomenon, all of these factors lead to difficulties in fruit identification and counting. Therefore, the accurate identification of citrus in complex scenes is highly important for informative yield estimation. For the above real scenarios, this study selects the most balanced YOLOv8n network, the latest detection networks YOLOv9t and YOLOv10n, and the new network YOLOv8-RL proposed in this paper for comparative analysis and detects citrus blossoms, green fruits, and ripening periods to explore the recognition ability of the new network in real environments in the field. The red circles in the figure indicate network leakage detection in different scenarios.

**Figure 8:**
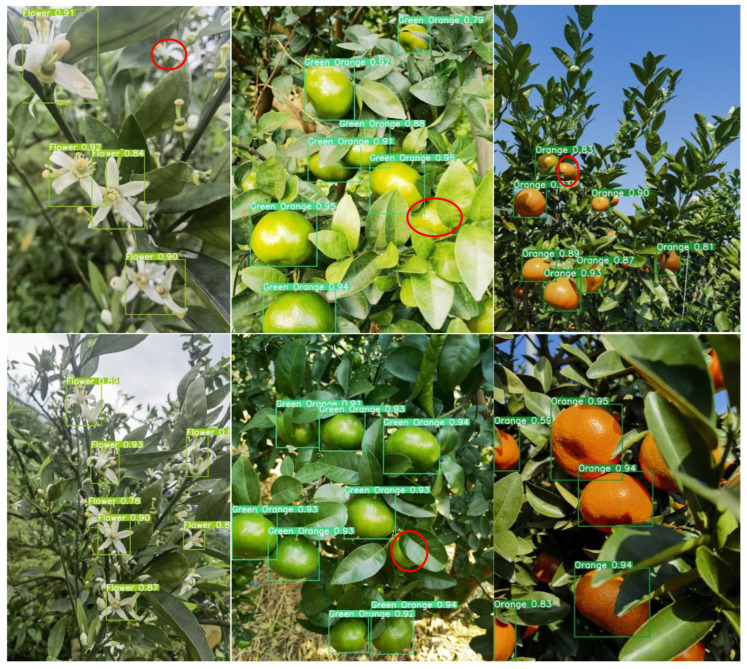
YOLOv8n detection situation.

As shown in Figure 8, the YOLOv8n network is able to recognize citrus targets effectively in all natural scenes. During the flowering period, citrus flower clusters are more densely distributed on the branches, and the leaves cover each other, increasing the network’s ability to recognize the flowers. Second, the network is not sensitive to the small targets at the edge of the image under backlight conditions, and there is a slight omission of detection. In the green fruit period, the network’s detection rate of the fruit is significantly improved. The analysis suggests that although the green fruit and green leaves are similar in color, the network has been trained to maintain the ability to judge the characteristics of the green fruit, which makes it possible to accurately identify the target even in the case of overlapping fruits and misshapen leaves and branches. As shown in the figure, the brightness of the light has a large effect on the detection ability of the network. In direct sunlight, the surface color of the green fruit is extremely similar to the color of the green leaves, and the network has a leakage detection ability. During the ripening period of citrus, the network has the highest detection rate of the fruit, and the analysis suggests that although the situation of fruit overlapping with leaf shade still exists, the citrus color features are obvious, and the network can effectively extract the texture features. Second, in the case of sufficient light, the network is more likely to obtain the color semantic information of the fruits, which improves the detection rate.

**Figure 9:**
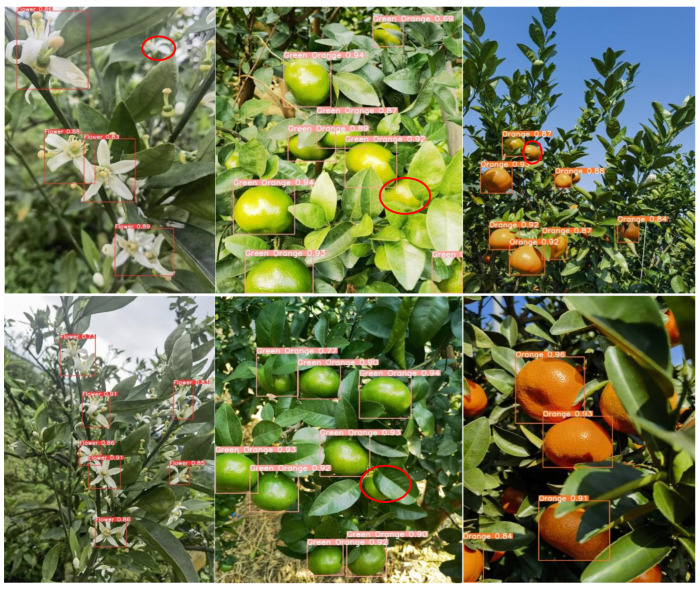
YOLOv9t detection situation.

**Figure 10:**
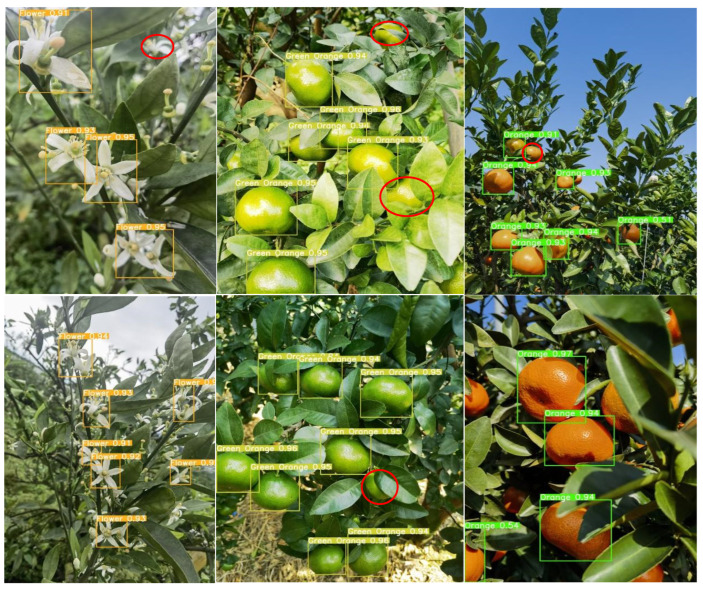
YOLOv10n detection situation.

Under the same detection scenario, both the YOLOv9t and YOLOv10n networks are effective at recognizing citrus full-growth images. As shown in Figure 9 and Figure 10, as the current state-of-the-art detection networks, the two are very close to each other in terms of their citrus detection ability. During the flowering period, individual flowers are all effectively detected, but in the case of leaf occlusion and flower sticking, the network loses the features of the target, and both of them have instances of missed detection. Second, during green fruit detection, the YOLOv9t detection rate was significantly greater than the YOLOv10n detection rate, and the number of green fruits detected was greater than the number detected in the same scenario; however, individual fruits were still not detected in direct light scenarios with interference from the background and the same color. During the citrus ripening period, the detection rates of the two networks remained the same, and the networks were able to recognize the fruits in both elevated and close-up images. In summary, as the latest target price detection networks, YOLOv9t and YOLOv10n exhibit excellent performance in terms of detection accuracy and the target detection rate alone, but the lower frame rate and large computational overhead are not suitable for the real-time detection of citrus in complex environments.

**Figure 11:**
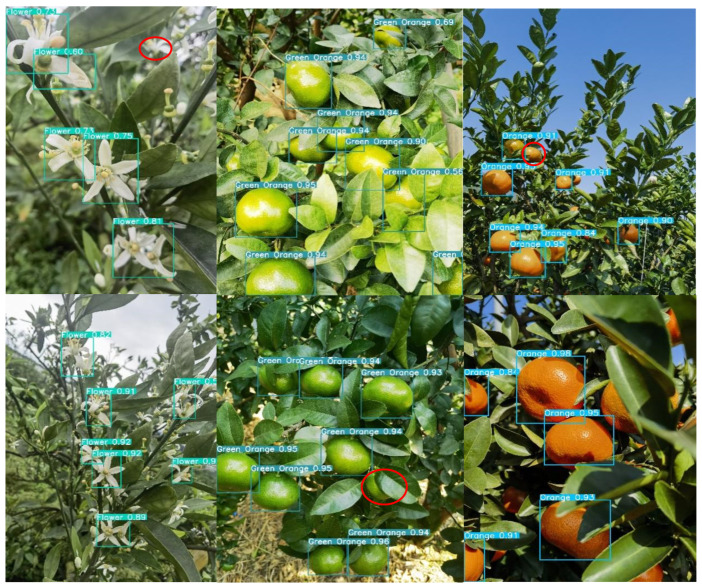
YOLOv8-RL detection situation.

In the actual field detection process, unavoidable factors such as light dimness, fruit overlap, and leaves covering each other present considerable challenges for fruit identification and counting. In practical detection, a network with high accuracy and light weight reduces the difficulty of real-time citrus monitoring and counting to a certain extent, as it can be deployed to portable devices such as mechanical vehicles or cell phones. The detection results of the YOLOv8-RL network proposed in this study in real scenarios are shown in Figure 11. With the number of parameters and the number of floating-point operations reduced to half of the original number, the recognition rate and target detection rate of the network are consistent with those of the YOLOv8n, YOLOv9t, and YOLOv10n networks. The lightweight network model can accurately identify the target at the flowering, fruiting, and ripening stages, which shows that the reduction in parameters does not affect the detection ability of the network and once again verifies the effectiveness of the network improvement, which is more suitable for real-time monitoring of citrus in complex scenarios.

### 4.3. Ablation Experiments

To again verify the effectiveness of the YOLOv8-RL network improvement strategy proposed in this study, an ablation experiment is set up to train 300 epochs in the same training set, and the optimal weights are selected for comprehensive evaluation in the same test set. The results are shown in Table 3. The analysis shows that after the network replaces only the C2f module with RGCSPELAN, the number of parameters and floating-point operations of the network decreases by a small amount, the model size is reduced to 4.7 MB, and the FPS value is improved by 6.4, which shows that the new module feature extraction and gradient circulation ability compensate for the loss of performance caused by the residual block well. The other modules were subsequently held constant, and only the detection head was replaced to test again. The results revealed that the number of network parameters of the detection head accounted for a large proportion of the total number of parameters in the YOLOv8n network, the number of network parameters decreased by approximately 25% by replacing only the detection head, the new detection head used the shared convolution method to reduce parameter redundancy while making target localization and classification more accurate, and the new detection head was more accurate in terms of P and mAP@.5 metrics than when the original network was improved by 1 percentage point while losing the frame rate advantage. On this basis, this study combines the advantages of the two modules by replacing both the C2f module and the detection head, and the results show that the new network further improves the model FPS value while keeping the number of parameters and computations further decreased and the accuracy unchanged, resulting in a more lightweight and real-time network.

### 4.4. Estimation Studies

To validate the usefulness of the YOLOv8-RL network, the study area of the Pengyu Brothers Citrus Demonstration Base in Gongcheng County was selected for field validation in this study. Sixteen citrus fruit trees were randomly selected from the orchard for yield estimation model construction and validation. Among them, 10 fruit trees were used for model construction, and 6 fruit trees were used for model validation. The detection network can recognize the number of fruits on one image, which provides a basis for predicting the number of fruits on a whole tree. Therefore, in this study, unilateral images of 16 fruit trees were collected during two growing periods, i.e., the green fruit stage and the citrus ripening stage, for the network to recognize the counts. The true number of fruits hanging on the tree was recorded visually by the human eye at the same time as the collection of the unilateral images, and the average of the three visual recordings was taken as the true value of the tree. A regression equation was then established between the true value of the tree’s fruits and the number of fruits recognized by the network, the number of fruits on the whole tree was obtained via local prediction of the whole, and the number of fruits on the whole tree was subsequently obtained [31]. Predicting the final number through the green fruit stage estimation model can achieve early estimation, and the maturity stage estimation model can effectively reduce the counting error and improve the accuracy of estimation.

#### 4.4.1. Estimation Modeling

**Figure 12:**
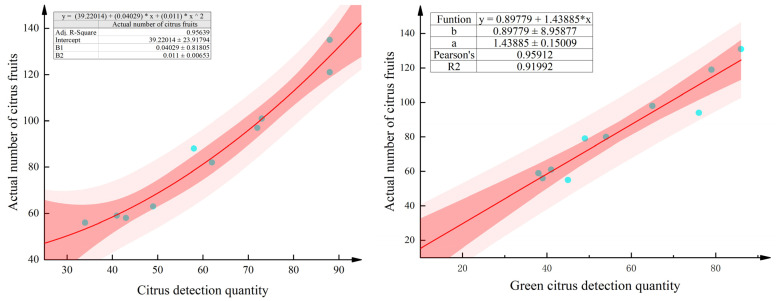
Plot of network counts fitted to the visualization of the human eye.

Table 4 and Table 5 show the number of network recognitions and the manual visual truth values of 10 fruit trees for two growth stages, the green fruit stage and the ripening stage, respectively. As shown in the table, the number of unilateral recognitions of each fruit tree is between 40 and 90, and the true value of the fruit tree is approximately 50–140. Unilateral fruit tree network identification is prone to leakage misdetection phenomena, identification of the number and true value of the gap, the analysis of the citrus orchard base reveals that the distance between the fruit tree distribution is not uniform, the branches and leaves of the fruit trees intersect each other, the fruits overlap with each other in terms of adhesion, and the degree of light dimming and other factors affect the network detection rate. The difference between the true values of different fruit trees is due to the randomness of fruit tree selection and fruit trees in terms of the growth process, light conditions, nutrients, soil moisture, and other external factors, as well as the yield of fruit trees [32]. Finally, the number of detections in the table was fitted to the true value to explore the relationship between the two. After linear and nonlinear fitting, the optimal fitting equation is shown in Figure 12. The results showed that during the green fruit stage, the optimal result was achieved when the fruit detection count was linearly fitted to the actual value, with a fitting equation of y = 1.43885x + 0.89779 and a determination coefficient R^2^ of 0.91992. During the mature stage, the optimal result was achieved when the fruit detection count was nonlinearly fitted to the actual value, with a fitting equation of y = 0.111 × ^2^ + 0.04029x + 39.22014 and a determination coefficient R^2^ of 0.95639, indicating a strong correlation between the recognized and true values.

#### 4.4.2. Validation and Analysis of Estimated Production Models

**Figure 13:**
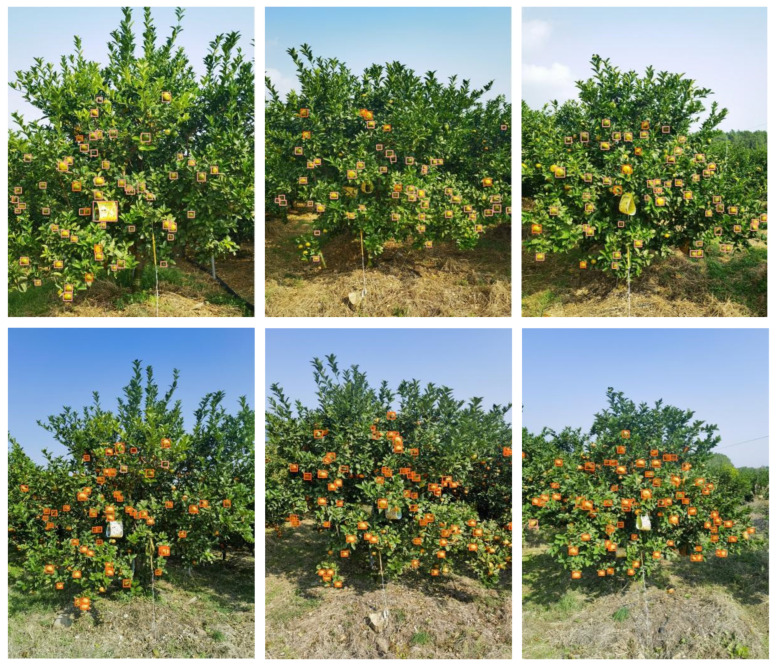
Unilateral identification of three fruit trees at the green and ripe fruiting stages.

To validate the applicability of the two estimation models in Section 4.4.1, in this study, six fruit trees were selected for model validation, and the true values of the citrus fruits were obtained via manual counting. The process is as follows: the side views of the six fruit trees in the green fruit stage and ripening stage were input into the detection network three times, the number of identifications was subsequently substituted into the estimation model for prediction, the average of the three detection results was taken as the detection value of the network, and the average of the three prediction values was taken as the final model prediction value. Figure 13 shows the results of the algorithm recognition of three of the fruit trees at the green fruit stage and maturity stage, from which it can be seen that the number of fruits in the side view of the complete fruit tree can roughly represent the overall yield of the tree.

The detected, true, and predicted values of the six citrus fruit trees mentioned above during the two growing periods are shown in Table 6 and Table 7, where the values are the average values of the fruit quantities obtained in the three ways. The absolute value of the difference between the true value of the fruit and the detected value is recorded as error E1, the absolute value of the difference between the true value of the fruit and the predicted value is recorded as error E2, and the percentage value of the error value of the true value is used as the error rate of the fruit quantity of the fruit tree. The results of the table analysis indicate that the same citrus fruit tree, green fruit stage, and maturity of the final number of fruits are very similar. In the analysis of the green fruit stage to promote the development of fruit, farmers prune the fruit tree or artificially control the number of fruits, resulting in the number of fruits in the green fruit stage not being very high. Second, regardless of the green fruit period or ripening period, the number of unilateral fruit tree images, the number of network recognition and the true value of the existence of a large error, as shown in Column E1 of the table, are the reasons that the network for the image of the unilateral detection of the results given is only the approximate number of fruit trees in one direction, many hidden in the branches and leaves of the fruit tree are one of the sources of error; the network in the detection of the overlap of the fruits and the foliage cover-up the phenomenon of omission detection is also a source of error; and the network in the detection of overlap and foliage cover-up generated by the fruits and leaves. The phenomenon of missed detection is also one of the sources of error. Therefore, using only the network to determine the number of counts as the final estimation of fruit tree yield results is more inaccurate.

On this basis, the predicted value of the fruit tree was obtained by substituting the network identification value into the estimation model, and the error between the predicted value and the true value was recalculated, as shown in Column E2. The number of errors in the two growing periods after correction by the estimation model is significantly smaller than the error generated by a single network detection count, and the accuracy of the prediction of the fruit yield can be further improved by using the estimation model. Second, the true error rate of the fruit counts corrected by the estimation model was calculated, and the results are shown in the columns of Table E2 errors, with an average error rate of 6.96% at the green fruit stage and an average error rate of 3.71% at the ripening stage. Although there are many fruits in the green fruit period, the network detection rate is low because of the similar color of fruits and green leaves, and the difference between the number of detections and the true value is large. The color of fruits in the ripening period is orange, and the detection network is more likely to learn its characteristics. The number of detections is close to the true value, and the coefficient of determination of the estimation model of citrus fruits in the ripening period is higher than that of the green fruit period, so the average error rate of the ripening period fruits after the correction of the estimation model is lower.

**Figure 14:**
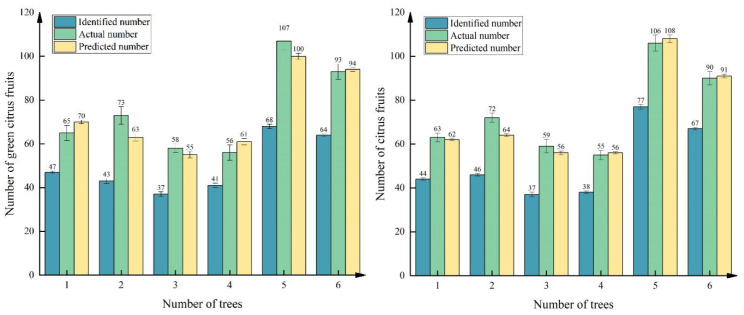
Histogram of fruit distribution at the green and mature stages of six fruit trees.

In summary, the fruit detection value, true value, and predicted value of six citrus fruit trees at the green fruit stage and maturity stage are shown in Figure 14, from which it can be seen that the error between the network identification value and the true value is larger, and it is not accurate to use only the network identification fruit count as the final fruit tree yield; after the correction of the two estimation models, the true value and the predicted value are much closer to each other, with lower error, which is more suitable for the estimation of fruit yield in complex environments. The estimation of fruit yield during the green fruit period can help farmers estimate fruit yield and income in advance and adjust the efficiency of agricultural production and resource allocation in time; the estimation of fruit yield during the ripening period is highly important for rationally arranging production and marketing plans and improving the quality of fruits.

## 5. Conclusions

In this paper, with citrus multigrowth period data as the data source, we propose a new network model, YOLOv8-RL, which is more lightweight and takes into account high accuracy, performs performance evaluation and complex scene detection comparison experiments, and conducts ablation experiments to verify the effectiveness of the new network improvement strategy. Then, on the basis of the new network recognition counting and manual field counting, we constructed a citrus green fruit stage and ripening stage estimation model, and utilized the estimation model for fruit prediction. On the basis of the experimental results, the following conclusions are drawn:

(1) On the basis of the citrus multiperiod dataset, the lightweight network model YOLOv8-RL proposed in this study reduces the number of model parameters and floating-point operations by approximately 50% compared with YOLOv8n, and the model size is only 3.2 MB. The average recognition rate of this network for citrus flowering, green fruit, and mature stages is 95.6%, the mAP@.5 is 94.6%, the inference time is only 2.3 ms, and the FPS frame rate is 123.1. It is lightweight while considering accuracy, which is helpful for deployment to edge devices with scarce computing resources for agricultural tasks and provides a reference for the design of lightweight networks.
(2) Compared with estimating yields with network recognition only, the two different growth period estimation models constructed on the basis of the new network model of recognition counts and manual counts had small errors and high prediction accuracies. The coefficient of determination R^2^ of the green fruit period estimation model is 0.91992, and the average error rate of fruit prediction is 6.96%. The coefficient of determination R^2^ of the ripening period estimation model is 0.95639, and the average error rate is 3.71%, which provides a theoretical basis for early yield prediction in citrus orchards and is important for current digitalized agriculture and monitoring of crop growth.

In this study, we conducted systematic research on citrus early yield estimation based on an improved target detection network using citrus multiperiod data, but further improvements are still needed. The next step is to deploy the lightweight network into agricultural equipment for field operations to realize the application value. The next step will focus on the problem of insufficient detection due to the adhesion of citrus flower clusters. It will construct the correspondence between citrus flowers and citrus fruits and establish the relationship between the number of flower clusters and the number of green fruits and orange fruits for the entire citrus tree throughout the whole. Additional steps will focus on the problem of insufficient detection due to flower cluster adhesion to construct the correspondence between the number of flower clusters and the number of green fruits and orange fruits during the entire growing period of the citrus tree to further improve the model of citrus early yield estimation and to realize more accurate early yield prediction.

## Figures and Tables

**Table 1:** Distribution of the datasets.

Dataset	Proportion	Images	Target Quantity
Training dataset	70%	2275	13,186
Validation dataset	10%	325	1941
Test dataset	20%	650	3778
Full dataset	100%	3250	18,905

**Table 2:** Comparison results of different target price detection network models.

Model	Parameter	GFlops	Size/mb	P/%	R/%	Map@.5/%	Inference/ms	FPS	F1
YOLOv5s	1,763,224	4.1	3.9	95.5	90.5	94.7	9	93.8	0.93
YOLOv7-tiny	6,013,008	13.0	12.3	97.0	92.5	96.6	9.5	92.5	0.95
YOLOv8n	3,157,200	8.9	6.6	95.4	91.2	94.5	3.4	118.1	0.93
YOLOv8s	11,126,745	28.4	22.5	97.9	92.5	96.9	8	101.8	0.95
YOLOv8m	25,841,497	78.7	52.1	98.2	93.3	97.1	19	42.3	0.96
YOLOv8l	43,608,921	164.8	87.7	98.3	94.9	98.0	70.4	25.0	0.97
YOLOv8x	68,126,457	257.4	136.7	98.5	94.4	98.1	182.3	14.9	0.97
YOLOv9t	2,617,730	10.7	6.1	95.5	91.5	95.9	28.8	32.5	0.94
YOLOv10n	2,695,586	8.2	5.8	94.5	90.2	94.5	13.2	69.4	0.93
Ours	1,553,846	4.5	3.2	95.6	89.8	94.6	2.3	123.1	0.93

Note: n/s/m/l/x represent the model sizes nano/small/medium/large/extra large, respectively; the YOLOv5 series is the smallest model; the bold font is the optimal value of the model detection index; mAP@.5 average accuracy when iou = 0.5.

**Table 3:** Comparison of ablation experiment indices. √ representing the use of this corresponding module.

Model	RGCSPELAN	LSCD	Para	GFlops	Size/mb	P/%	mAP@.5/%	FPS	F1
YOLOv8			3,157,200	8.9	6.6	95.4	94.5	118.1	0.93
	√		2,197,561	6.0	4.7	95.8	94.3	124.5	0.93
		√	2,362,518	6.5	5.0	96.4	95.4	117.2	0.94
	√	√	1,553,846	4.5	3.2	95.6	94.6	123.1	0.93

**Table 4:** Network identification and true value statistics of the citrus green fruit stage.

Class	Detect	Actual	Class	Detect	Actual
1	45	55	6	79	119
2	41	61	7	86	131
3	54	80	8	76	94
4	49	79	9	38	59
5	65	98	10	39	56

**Table 5:** Citrus ripening period network identification and true value statistics.

Class	Detect	Actual	Class	Detect	Actual
1	43	58	6	88	121
2	49	63	7	88	135
3	62	82	8	72	97
4	58	88	9	34	56
5	73	101	10	41	59

**Table 6:** Yield prediction error statistics in the citrus green fruit stage for six fruit trees.

Number	Detect	Actual	Predicted	E1	E2	E2 Errors
1	47	65	70	18	5	7.6%
2	43	73	63	30	10	13.6%
3	37	58	55	21	3	5.1%
4	42	56	61	14	5	8%
5	68	107	100	39	7	6.5%
6	64	93	94	29	1	1.0%

**Table 7:** Yield prediction error statistics in the citrus ripening stage for six fruit trees.

Number	Detect	Actual	Predicted	E1	E2	E2 Errors
1	44	63	62	19	1	1.5%
2	46	72	64	26	8	11.1%
3	37	59	56	22	3	5.0%
4	38	55	56	17	1	1.8%
5	77	106	108	29	2	1.8%
6	67	90	91	23	1	1.1%

## Data Availability

Data will be made available on request.
